# Use of an Immersive Virtual Reality Application to Educate Medical Students in Patient Handover: Pilot Study

**DOI:** 10.2196/73907

**Published:** 2025-08-27

**Authors:** Laura Isabel Hanke, Patrick Schwoerer, Florentine Huettl, Lukas Vradelis, Kai-Uwe Strelow, Christian Boedecker, Patrick Saalfeld, Vuthea Chheang, Holger Buggenhagen, Hauke Lang, Christian Hansen, Tobias Huber

**Affiliations:** 1 Department for General, Visceral and Tranplant Surgery, University Medical Center of the Johannes Gutenberg-University Mainz, Langenbeckstr. 1, Mainz, 55131, Germany, 49 6131 17 7291; 2 Rudolf Frey Educational Clinic, University Medical Center of the Johannes Gutenberg-University Mainz, Mainz, Germany; 3 Virtual and Augmented Reality Group, Faculty of Computer Science, Otto-von-Guericke University Magdeburg, Magdeburg, Germany

**Keywords:** immersive virtual reality, medical education, head-mounted display, handover, gamification, new tools

## Abstract

**Background:**

Patient handover is a daily task for doctors and nurses, and structured handovers have been proven to positively impact patient outcomes. To teach the handover procedure, different communication tools have been applied, such as the ISBAR (introduction and identification, situation, background, assessment and actions, and recommendation) method.

**Objective:**

This study aimed to assess the effectiveness and user engagement of the first-time use of supplementary handover training in virtual reality (VR) for medical students as an addition to an existing curriculum. Furthermore, the VR program was tested for its usability, immersion, visually induced motion sickness (VIMS), and eye strain. Participants were evaluated for their motivation, time spent studying, and experience in VR, as well as their impressions of the use of VR in medical education.

**Methods:**

Handover training using the ISBAR method and patient actors is part of the curriculum in surgery of the eighth semester of human medicine studies in Mainz. Knowledge is tested via an Objective Structured Clinical Examination (OSCE) using patient actors. We developed an immersive VR application using 360° video surroundings with structured patient cases. This application was offered as an optional supplementary training in groups of three with a peer tutor. Parameters evaluated included participants’ characteristics, usability, and VIMS. Furthermore, a survey of the entire semester was conducted regarding their experience using VR and their enjoyment of studying. Finally, OSCE scores were collected and compared between the groups.

**Results:**

The study was conducted over two semesters, and 92 of 385 (23.9%) volunteering students were recruited. The median age was 25 (IQR 23-25) years, and the majority were female (n=61, 68.5%). There were few to no issues regarding VIMS and eye strain (median eye strain 1, IQR 1-2; median VIMS 1, IQR 1-2). There was no significant difference in students’ motivation (mid rank participant 107.84; mid rank nonparticipant 122.61; *P*=.11) and the amount studied for the subject (mid rank participant 113.88; mid rank nonparticipants 119.42; *P*=.54). Students felt significantly more confident in patient handover after the additional training (7-point Likert scale; mean pretraining 3.96, SD 1.39; mean post-training 3.17, SD 1.41; *P*<.01) and reported significantly more fun studying than their peers who did not participate in the additional training (mean participants 2.8, SD 1.54; mean nonparticipant 3.69, SD 1.73; *P*<.01). OSCE scores did not differ between the groups (median score 17 in both groups, IQR participants 16-19; IQR nonparticipants 16-18; *P*=.62).

**Conclusions:**

This study shows that applications in VR, if implemented in a structured curriculum, can be a helpful and safe addition to the teaching of communication skills. VR applications should be considered as a time-flexible, safe, fun, and motivating educational tool as an addition to curricular teaching.

## Introduction

Patient handover is a routine and essential component of daily clinical practice in hospitals across the world. Regardless of whether inpatient or outpatient, elective or emergency care, all departments involved in patient care must regularly conduct handovers of patient cases. This involves all medical staff and includes intra- and interprofessional as well as intra- and interdisciplinary communication [1].

**Figure 1.**
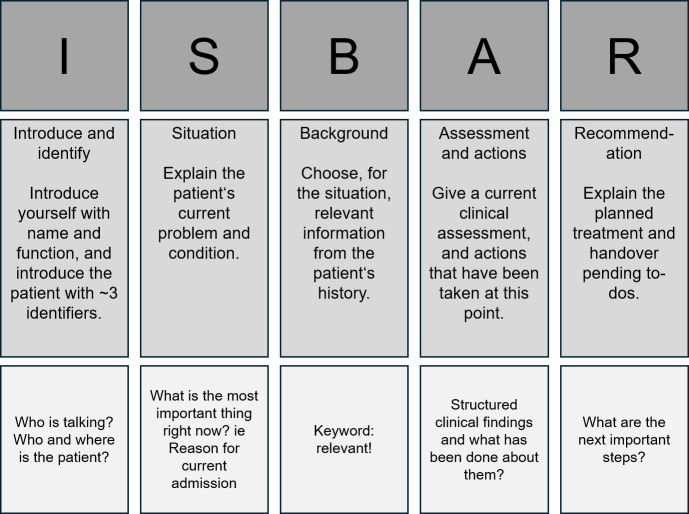
Introduction and identification, situation, background, assessment and actions, and recommendation (ISBAR) method in schematic form. Row 1: abbreviation, row 2: title and description, and row 3: additional cues.

It has been shown that patient handover is a key element for adequate patient care, directly influencing patient outcomes and yielding an important aspect of patient safety [2,3]. To facilitate comprehension by the receiving party, the handover should follow a clear structure, use concise language, and focus on essential information. Furthermore, patient handover integrates a variety of communication skills into one task that can be taught and practiced. In response to this need, the ISBAR (introduction and identification, situation, background, assessment and actions, and recommendation) framework was developed, tested, and validated across different medical professions [4-6]. ISBAR provides a structured backbone for every handover situation [7]. The details of the ISBAR concept are presented in Figure 1*.*

The teaching of communication skills is essential for all medical professionals and has been increasingly incorporated over the last few decades [8]. In Germany, clear learning objectives for communication have been implemented in medical licensing regulations, posing new challenges for educators and academic institutions [9]. Most medical professionals practicing today did not receive formal communication training until years into their careers, if at all, because such training was not emphasized during their undergraduate and postgraduate education [10]. As a result, a crucial first step is to train the trainers, because good clinical expertise does not automatically translate into teaching competence [11].

Moreover, traditional courses, lectures, and written examinations are often ill-suited for communication training. Effective communication training requires a good balance of theoretical knowledge, practical application, self-reflection, and feedback from both instructors and peers. A sound theoretical basis is indispensable to ensure reproducible and consistent results. However, practical skills can only be trained effectively in practical courses where learners actively apply their newly acquired competencies [8].

This experiential learning setting is frequently supported by employing actors as simulated patients, which provides a valuable but costly asset [12].

Self-reflection is key to learning communication skills. This might take dedicated time in or after class to reflect individually or in a small group on why certain situations proved challenging to handle or why some interactions were perceived to be more stressful than others. Feedback from instructors and peers is important not only to correct theoretical and contextual shortcomings but also to raise awareness of how one is perceived by others. Many communication classes incorporate video reviews, allowing students to observe and evaluate their own behavior from an external perspective [13,14].

Applying the principle of constructive alignment, a practical skill such as patient handover cannot be adequately tested using multiple-choice or written examinations, but should instead be tested through an Objective Structured Clinical Examination (OSCE) [15]. This style of examination has been successfully used in the medical field for years [16]. In contrast to a written examination, which hundreds of students can take simultaneously, the OSCE requires students to rotate through different stations and complete each task individually. Consequently, OSCEs are more time-consuming and inherently more resource-intensive.

In recent years, virtual reality (VR) technology using head-mounted displays has become easily accessible to the public, especially in recreational computer science, and has also seen widespread use in different research settings. Among its many applications, VR presents an interesting addition to educational programs, facilitating learning in a safe environment at the learner’s own pace and schedule [17]. Furthermore, learning in VR has been reported to be enjoyable for students and capable of fostering autobiographical memory, which may improve knowledge retention and recall [18]. VR applications have been developed for procedural and communication skill training [19].

In recent years, VR has gained additional interest due to its capacity to bridge the gap in situations where direct contact with patients or peers was not feasible, as experienced during the global COVID-19 pandemic [20]. While VR has not yet been able to replace interactions with peers, patients, and teachers, it is steadily evolving into a useful tool to enrich educational programs [17,21,22].

This study aimed to develop and test a supplemental educational tool for training patient handover in VR within a curricular course for medical students.

## Methods

### Study Setting and Educational Concept Design

At the University Medical Center of Johannes Gutenberg-University Mainz, Germany, communication skills are taught through a longitudinal communication curriculum encompassing various disciplines over multiple semesters. One component of the curriculum is teaching patient handover within the framework of the Surgical Practical Course (SPC), which takes place at the beginning of the 8th semester.

In this unit, students are taught the ISBAR concept through a short theoretical overview followed by practical training with standardized patient actors. They receive feedback from both instructors and peers. The unit lasts 45 minutes and involves small groups of 4-6 students. In addition, videos and texts are made available online for review. At the end of the semester, students undergo an OSCE using patient actors, complying with the concept of constructive alignment [15].

While other components of the SPC, such as surgical knot tying or suturing, can be practiced independently at home or in a skills laboratory, patient handover is more challenging to practice. To address this limitation, we developed a VR training suite consisting of three cases with increasing urgency. The 3 scenarios involve patient actors and SPC instructors as actors and were recorded. They are embedded in a structured curriculum to complement the VR training suite.

**Figure 2.**
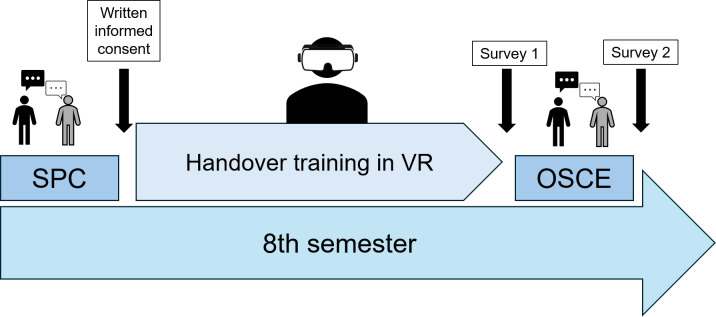
Course of the study over one semester. In the first week of the semester, the Surgical Practical Course (SPC) took place, including the subject of patient handover taught with patient actors. Over the course of the semester, there were multiple time slots for a one-time handover training in VR. In the last week of the semester, the Objective Structured Clinical Examination (OSCE) took place, including examination of the subject of patient handover using patient actors. Black arrows indicate time points of informed consent and survey of participants and nonparticipants in the same semester. OSCE: Objective Structured Clinical Examination; SPC: Surgical Practical Course; VR: virtual reality.

During VR training, students progress through each scenario by answering short multiple-choice questions to obtain a patient’s history. They also receive a data sheet similar to a patient chart. The training sessions in VR are conducted in groups of three, each led by a peer instructor, thus enabling each student to perform one scenario. The training took place in the newly established VR training center at the Rudolf-Frey-Lernklinik for the semester. Figure 2 provides an overview of the course of the study.

### Scenario Design and Video Production

An interdisciplinary team conceptualized scenarios involving the same preoperative patient undergoing Warfarin therapy before inguinal hernia repair, with an increasingly urgent need for medical attention. Detailed scripts for all scenarios are provided in Multimedia Appendix 1.

#### Scenario 1

The participant arrives in the patient’s room and tells the participant that he has continued his daily medication of Warfarin until this morning, despite undergoing inguinal hernia repair tomorrow. Shortly thereafter, the attending physician arrives for the handover.

#### Scenario 2

The participant arrives in the patient’s room, who tells the participant that he has continued his daily medication of Warfarin up until this morning and has noticed black stools in the past few days. He also reports feeling faint. As additional information, the participant is given laboratory results with decreased hemoglobin levels. Shortly thereafter, the attending physician arrives for the handover.

#### Scenario 3

The nurse calls the participant to assist her because the patient has fainted, and his bed is covered in bloody stool. His blood pressure is low and heart rate elevated. Shortly thereafter, the medical emergency team and the attending physician arrive in the room for a handover.

The participants perform a quick history and then give a structured handover to the arriving doctor using the ISBAR concept. A detailed script of the scenarios is provided in the Multimedia Appendix 1. The videos were recorded in 8k resolution using a 360° camera (Insta360 Pro 2; Insta360) in an actual patient room. The decision to use highly immersive video footage with actors in an actual hospital setting, rather than animated surroundings, was supported by prior research, which showed that participants preferred more realistic scenarios [23]. The shoot was supported by the company Visual-Impressions, which specialized in 360° video shooting (Visual-Impressions GmbH).

### Development of the 360° Immersive VR Environment

**Figure 3.**
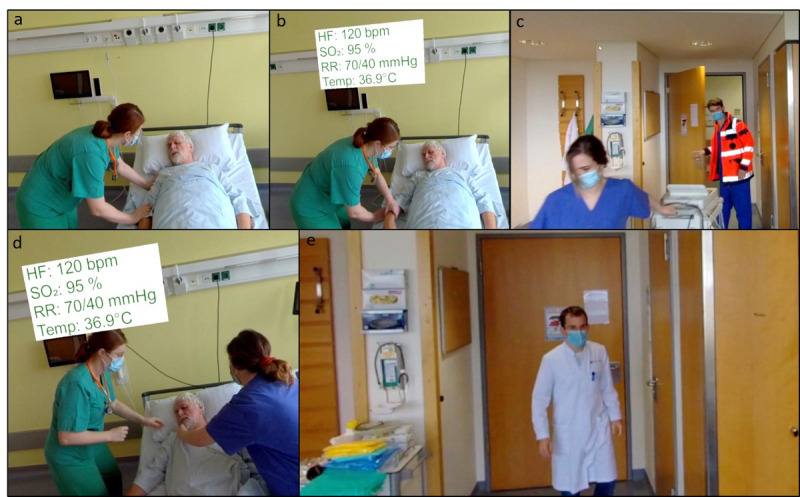
Screenshots of the virtual reality (VR) environment. In (A), a nurse finds the patient, who feels dizzy and unwell. In (B), she checks his vital signs, which are depicted in the box above her head. In (C), the emergency team arrives. In (D), the patient is connected to monitoring. In (E), the attending arrives to receive the patient handover. HF: heart frequency; RR: blood pressure; SO_2_: oxygen saturation; Temp: temperature.

We used the game engine Unity (version 2019.2.18f1; Unity Technologies) for an immersive VR development environment. The Virtual Reality Toolkit (version 3.3.0; Unity) was used to provide basic user interactions in VR, including locomotion and user interface (UI) interactions. The recorded 360° videos were edited and stitched according to the scenarios using the Shotcut (Meltytech, LLC) video editing tool. The stitched videos resulted in an equirectangular format that can be used for spherical projection mapping in VR. We incorporated the VIVE Media Decoder (HTC Vive Pro), a powerful video decoding plug-in, to achieve high-quality video streaming in Unity. The VR headset used for this study was the HTC Vive Pro (HTC Vive Pro), with a resolution of 1440×1600 pixels per eye (2880×1600 pixels combined), a refresh rate of 90 Hz, and a field of view of 110 degrees. Additional information regarding the scenarios was integrated under close medical and educational supervision and displayed on UI panels in VR. Participants were able to use their controllers with the ray-casting technique to interact with the UI interfaces. We also implemented an interactive questionnaire using the Virtual Reality Questionnaire Toolkit, allowing participants to answer questions directly in VR. If the participant answered the question correctly, the UI panel turned green and the scenario continued. If answered incorrectly, the scenario stopped at the question until it was answered correctly. Unlimited attempts were allowed. Figure 3 provides an impression of the VR environment.

### Recruiting and Data Collection

Volunteer participants were recruited over two semesters (summer semester 2023 and winter semester 2023/2024). Participants underwent VR training in groups of three, together with a student teacher, and received corrections and feedback after each handover from their peers and the teacher. Students were made aware of the study via email and personal communication during the curricular SPC, and volunteers were recruited after the end of the SPC and throughout the semester. Participants were asked to complete questionnaires at the end of the VR training regarding demographic information, personal motivation, and the Immersion Scale according to Nichols et al [24], as well as rating how confident they were in the subject of patient handover. Furthermore, data regarding visually induced motion sickness (VIMS) and eye strain were collected [25]. At the end of the semester, after completion of the OSCE, participants were questioned again regarding motivation, confidence, and preparedness for the OSCE, as well as their impression of VR as a useful tool for education. Participants were contacted directly via email for the second survey and reminded once. In addition, the entire semester was questioned as a control group. The OSCE results for the handover examination and overall OSCE results were recorded for analysis. For data collection, the online platform LimeSurvey (LimeSurvey GmbH, open source) was used. Questionnaires, in addition to the Immersion Scale by Nichols et al [24], were custom-made for this study and have not been published previously. The custom-made questionnaires are available in Multimedia Appendix 2.

### Statistical Analysis

Statistical analyses were performed using SPSS (version 29.0; IBM SPSS Statistics). Participant characteristics were analyzed using descriptive parameters. Questionnaire items were analyzed using the Mann-Whitney *U* test and *t* tests, as appropriate.

### Ethical Considerations

All participants were volunteer medical students in the 8th semester of the human medicine program at Johannes Gutenberg-University Mainz, enrolled in the SPC. Written informed consent was obtained at the beginning of the study. Participation was not reported to OSCE examiners or any other teaching personnel. Ethical approval was obtained under the reference code 2023‐17025–andere Forschung erstvotierend from the ethics committee of the regional medical association of Rhineland-Palatinate (Landesärztekammer Rheinland-Pfalz). Participants did not receive compensation. People visible in pictures (ie, Figure 3) are paid actors or coauthors and have provided written consent for publication.

## Results

Overall, 92 participants of 385 (23.9%) students were recruited for the study, of which 3 participants (amounting to one group) dropped out because of exceptionally bad weather conditions, which made them unable to attend training. After obtaining written informed consent, the participants were divided into groups of three and attended VR training. Data analysis was performed on 89 participants who attended training (per-protocol analysis). The median age of the participants was 25 years (IQR 23-28), and the groups consisted of 61 (68.5%) female and 28 (31.5%) male participants. In total, 40% (n=36) of the participants had prior experience in the medical field (ie, nurses, physical therapists, and paramedics). The second survey, after the OSCE, was completed by 81 of the 89 (91%) participants.

**Figure 4.**
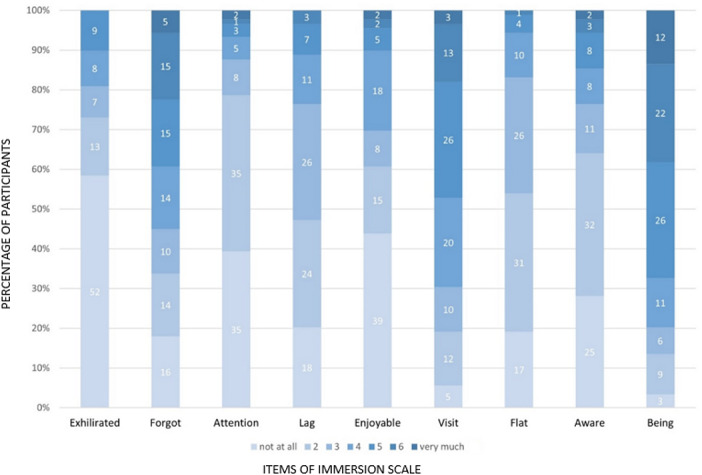
Result of the Immersion Scale as described by Nichols et al, based on a 7-point Likert scale. The questions of the scale are displayed with abbreviated titles; results range from 1 (not at all, at the bottom of the graph in lighter colors) to 7 (very much, at the top of the graph in darker colors). The results show a high grade of presence with minimal lag and minimal exhilaration, indicating a high grade of immersion.

Most participants had minimal to no experience using VR equipment or head-mounted displays (never: n=49, 55.1%; <5 times: n=33, 37.1%). Eye strain and VIMS were reported to be minimal, with one singular outlier who was able to complete the training despite dizziness (median eye strain 1, IQR 1-2, min 1, max 9; median VIMS 1, IQR 1-2, min 1, max 16). The majority of participants stated they felt present in the virtual world (scale of 1‐7, 1=not at all, 7=very much; n [5 or more]=60, 67.4%). Details of the Immersion Scale Questionnaire results are shown in Figure 4*.*

**Figure 5.**
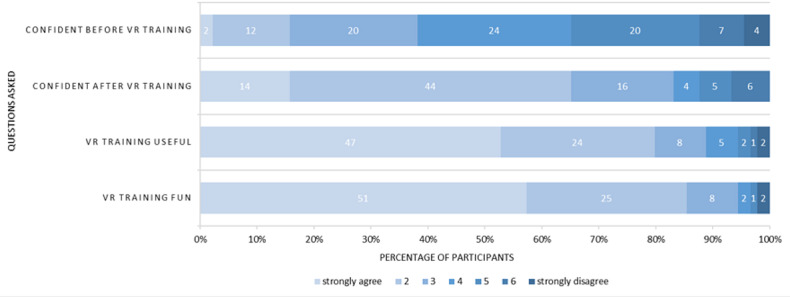
Results of pre- and post-training questionnaires. Short-form questions are displayed on the left, and answers are listed from left to right, starting with “strongly agree” (lighter colors) and ending with “strongly disagree” (darker colors). Rows 1 and 2: participants felt significantly more confident in giving a patient handover after VR training (mean pretraining 3.96, SD 1.39; mean post-training 3.17, SD 1.41; *P*<.001). Rows 3 and 4: after experiencing the training, they deemed virtual reality (VR) programs for medical education to be useful and stated that they had more fun in the VR training than in other courses (7-point Likert scale, 1=strongly agree, 7=strongly disagree; mean useful 1.56, SD 1.34; mean fun 1.71, SD 1.16).

Participants reported feeling significantly more confident in giving a patient handover after the training (median pretraining 4, IQR 3-5; median post-training 3, IQR 2-4; *P*<.001). The majority stated that they enjoyed the handover training in VR and had more fun than in other courses during the training (scale 1‐7; 1=a lot more fun and enjoyable than other courses; 7=a lot less fun and enjoyable; n (1)=47; 52.8%, n (2)=24; 27%). Further results are shown in Figure 5*.*

**Figure 6.**
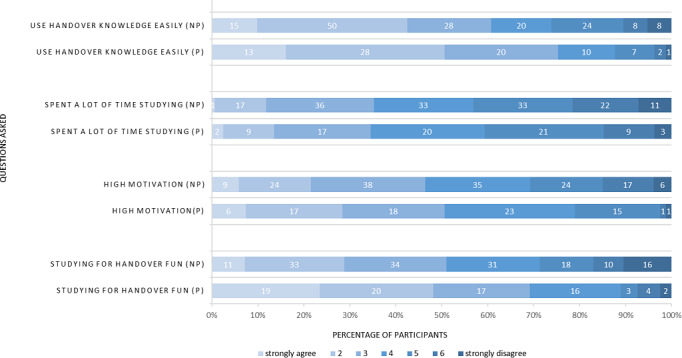
Comparison of nonparticipants and participant responses in the second survey. Short-form questions are displayed on the left, a 7-point Likert scale, answers are listed from left to right, starting with strongly agree (lighter colors) and ending with strongly disagree (darker colors).

A total of 153 of 296 (51.7%) students from the entire semester who had not participated in the training replied to the survey. When comparing survey results between VR-training participants and nonparticipants, a few significant differences were noted. Students were asked if they felt they were able to recall and use what they had learned about patient handover well in the OSCE (scale 1‐7; 1=very much, 7=not at all; mean participant 2.75, SD 1.36; mean nonparticipant 3.29, SD 1.65; middle rank participant 104.38; middle rank nonparticipant 124.45; *P*=.03; *r*=0.14). When asked whether they had fun studying for the patient handover, participants stated to have had significantly more fun (scale 1‐7; 1=very much, 7=not at all). Mean participant 2.8, SD 1.54; mean nonparticipant 3.69, SD 1.73; middle rank participant 94.82; middle rank nonparticipant 129.51; *P*<.001; *r*=0.25. Having experienced the training, significantly more participants deemed VR equipment and head-mounted displays as a useful supplement for medical education (scale 1‐7; 1=very much, 7=not at all; mean participant 1.56, SD 0.96; mean nonparticipant 3.03, SD 1.62; middle rank participant 54.98; middle rank nonparticipant 110.73; *P*<.001; *r*=0.42). It should be noted that all students were highly motivated (scale 1‐7; 1=very much, 7=not at all), and no significant difference in motivation was observed between groups (mean participant 3.38, SD 1.31; mean nonparticipant 3.76, SD 1.52; middle rank participant 107.84; middle rank nonparticipant 122.61; *P*=.11; *r*=0.11). Further results of the second survey are shown in Figure 6: (1) rows 1 and 2: participants stated more often than nonparticipants that they were able to recall and apply what they had learned about patient handover in the OSCE (mean participant 2.75, SD 1.36; mean nonparticipant 3.29, SD 1.65; middle rank participant 104.38; middle rank nonparticipant 124.45; *P*=.03; *r*=0.14), (2) rows 3 and 4: participants and nonparticipants spent a similar amount of time studying for patient handover, with no significant difference between groups (mean participants 4.09, SD 1.39; mean nonparticipants 4.24, SD 1.46; middle rank participants 113.88; middle rank nonparticipants 119.42; *P*=.54; *r*=0.04), (3) rows 5 and 6: motivation between participants and nonparticipants was not significantly different (mean participants 3.38, SD 1.31; mean nonparticipants 3.76, SD 1.52; middle rank participants 107.84; middle rank nonparticipants 122.61; *P*=.11; r=0.11), (4) rows 7 and 8: p stated to have significantly more fun than np when studying about patient handover (mean participants 2.8, SD 1.54; mean nonparticipants 3.69, SD 1.73; middle rank participants 94.82; middle rank nonparticipants 129.51; *P*<.001; *r*=0.25).

It should be noted that there was no significant difference in OSCE results, neither in the handover station nor in the overall grade (median grade participants 17, IQR 16-19; median grade nonparticipants 17, IQR 16-18; *P*=.62), with a small effect size (Cohen *d*=0.014).

## Discussion

### Principal Findings

Patient handover is an integral component of clinical practice that directly influences patient outcomes. The ISBAR method provides a clear structure that is easily remembered and reproducible [7]. However, the development of practical skills requires hands-on training, which can be time- and cost-intensive [8].

This study presents a novel VR-based training program for patient handover in a surgical context, which is tailored to our didactic framework and can be easily used with a student tutor. Interest in the study was high, and participant recruitment proceeded rapidly. When asked about their motivation to participate, most students stated that they were curious to explore innovative technologies and learning methods.

Our findings indicate that the VR training program was perceived as an engaging and enjoyable addition to other educational concepts, without drawbacks such as severe VIMS or eye strain. While there was no significant effect on OSCE results, which is consistent with prior study results [26], participants felt more confident after handover training in VR. This is particularly interesting in the context of communication skills, as confidence can play an important role in skill demonstration and application.

### Comparison With Prior Work

To the authors’ knowledge, there are no similar studies or programs using 360° immersive VR in a curricular setting currently available. In 2023, Andreasen et al [27] compared a VR tool with traditional paper-based learning methods. However, the study used a desktop VR application without head-mounted displays or a fully immersive 360° environment. Furthermore, the evaluation between groups was conducted using a written test. We deliberately decided against this approach to respect the concept of constructive alignment [15]. In the past, our group was able to show that VR simulation using 360° immersive video is preferred by participants; hence, we chose this mode of demonstration over others [28]. Similar to our results, the authors described similar outcomes in the examination, but students using the VR application reported finding this way of learning more enjoyable [27].

Neher et al [29] presented a feasibility study of interprofessional team training for medical and nursing students in VR in 2024. While this study uses immersive VR with head-mounted displays, it is a computer-generated environment in contrast to the program we are presenting. As this was a feasibility study, there was no implementation in curricular teaching or comparison with a control group, as in our study.

Prior studies have evaluated the teaching of structured patient handovers in theoretical form, which mostly includes lectures and observation of handovers in video material with subsequent group discussion [30,31]. Darbyshire et al [32] used structured teaching materials and group discussions of videos, with high satisfaction among students, even though there were no practical exercises involved. Gordon et al [33] and Thaeter et al [34] added role-play scenarios to the theoretical teaching approach to facilitate practice. All three aforementioned studies differ from the presented paper not only in the use of VR but also in the fact that there was no curricular teaching of patient handover, and the handover courses offered were optional and not graded. Thaeter et al [34] compared students who completed the handover course to a control group with no handover teaching at all and found an expectedly high statistical significance, which cannot be reached with a purely supplemental teaching effort, as described in the presented paper.

However, simulation can be achieved not only in VR but also with simulated patients, as is used in the SPC described. Simulated patients are effectively used in medical education, mainly for training communication skills [35]. Losfeld et al [36] were able to show that handover curricula can successfully be augmented with e-learning in a blended learning concept. To the best of our knowledge, there is currently no handover curriculum comparing peer-to-peer role-play with simulated patients and VR simulation, which would certainly be an interesting topic for future research.

It should be noted that studies on handover naturally include all medical professions and curricula, and study designs and approaches may differ between different target groups.

### Limitations

Although our study yielded interesting results, several limitations should be acknowledged. First, studies that rely on voluntary participation, such as ours, tend to attract the interest of highly motivated students, potentially introducing selection bias in the results. These students tend to exhibit greater intrinsic motivation and engage more intensely with teachers and learning materials than others do. To assess this, we specifically asked both participants and nonparticipants about their motivation and time invested in studying. No significant differences were found between the groups in terms of self-reported motivation or time spent preparing for the patient handover. This suggests that the selection bias between groups was minimal; however, as these measures were based on self-assessment, their validity may be limited.

Second, our study compared additional VR training with no additional training. A valuable direction for future studies could be the inclusion of a third group receiving additional training with standardized patient actors. We deliberately chose not to add more theoretical learning materials (ie, written case studies or video footage), since this would have shifted the focus to a comparison between practical and theoretical teaching modalities.

Third, the OSCE itself may present a limiting factor. The OSCE results were overall very good for all stations, with most students receiving very good grades and no examination failures requiring repetition. This raises the possibility that the OSCE lacked sufficient discriminatory power to detect differences in performance because all students did well on the examination. Nonetheless, the strong alignment between the SPC and the OSCE emphasizes the high degree of constructive alignment in the curriculum—an important feature for teaching quality, even if it complicates research evaluation [15].

### Future Directions

Although handover training in VR was perceived well by participants, it still required students to attend in-person training sessions at the University Medical Center Mainz, Germany. While the availability of multiple time slots provided greater flexibility compared to training with standardized patients, the program does not yet offer true on-demand accessibility, such as an at-home application. Achieving this level of flexibility in communication training remains challenging, as peer and tutor feedback are critical components of skill development. In the future, applications may be able to assess human communication and provide immediate feedback. In the near term, however, this feedback will most likely be limited to the assessment of correct and complete content and the order of information in line with the ISBAR format. During the handover training sessions, students were enthusiastic about the VR environment and provided valuable feedback for future renditions of the program. A common request was for more time to practice, including access to additional scenarios as well as the opportunity to repeat existing ones.

Future research is currently directed at the implementation of different scenarios; yet, technological advances such as the integration of speech recognition and natural language processing tools to enable automated assessment of handover performance will need further exploration. Implementing such a system would require substantial redevelopment and technical sophistication, but it represents a promising area of development. The evaluation of more advanced features, such as tone, facial expressions, and other nonverbal aspects of communication, would require highly sophisticated technical solutions that are not yet widely available [37,38]. These additions could potentially facilitate communication training unconstrained by time or instructor availability and will certainly be subjects of future research.

However, it should be critically evaluated whether communication training without human interaction is a goal we are trying to achieve. Although the VR program was positively received, students stated that they would not prefer training in VR over bedside teaching. The authors fully support this sentiment: contact with patients and peers should not be replaced, but rather enhanced by technological innovations.

### Conclusions

Overall, our study was able to show that VR communication training can be a valuable addition to conventional educational programs, leading to students feeling confident in giving a patient handover. While additional teaching programs in VR can be an interesting and motivating supplementary aid, they are not able to replace current teaching methods, especially bedside teaching. VR applications should be considered as a time-flexible, safe, and fun educational tool to supplement curricular teaching.

## Supplementary material

10.2196/73907 Multimedia Appendix 1 Scripts for virtual reality scenarios.

10.2196/73907 Multimedia Appendix 2 Translated questionnaires.

## Abbreviations

ISBAR: introduction and identification, situation, background, assessment and actions, and recommendation
OSCE: Objective Structured Clinical Examination
SPC: Surgical Practical Course
UI: user interface
VIMS: visually induced motion sickness
VR: virtual reality
