# Supplementary material for: Use of an Immersive Virtual Reality Application to Educate Medical Students in Patient Handover: Pilot Study
Source: JMIR Serious Games. 2025 Aug 27;13:e73907. doi: 10.2196/73907 (PMC12384679; doi:10.2196/73907)
Supplement: Multimedia Appendix 1 [file games-v13-e73907-s001.docx]

Script for VR scenarios for the manuscript “Use of an immersive virtual reality application to educate medical students in patient handover”

**Patient history:**

Name: Willi Karstens

Date of Birth: 27.11.1950

Main concern: Inguinal hernia with increasing discomfort and pain when moving, Scheduled for elective open hernia repair.

Further, preexisting illnesses and conditions

- Atrial fibrillation with absolute tachyarrhythmia, medicated with Marcumar
- Arterial hypertension, treated with Ramipril
- Hyperlipidemia, treated with Simvastatin
- Benign prostate hyperplasia
- S.p. laparoscopic appendectomy 2008
- Nicotine use, 40 pack years
- Alcohol use, 2 bottles of beer/day
- Additional medication: ASS 100, Ibuprofen (as needed)

**Scenario 1**

*Nurse approaches the patient, who is in his room in a hospital bed.*

*Nurse:* “Good morning Mr. Karstens, this is our intern, who will draw a blood sample now. We have to check up your INR before operation, which was still elevated.”

*Nurse points towards participant, while introducing them.*

*Patient:* “Good Morning, I would like to ask you a question, if I may. I took my Marcumar pills up until last night but I forgot to bring them. Can you provide me with my medication?”

*Nurse:* “You were not supposed to take your Marcumar for 5 days prior to the operation, because it is a blood thinner. Were you not aware about this? It is possible that we have to postpone the operation now.”

*Attending enters and greets everybody.*

*Attending:* “Good Morning Mr. Karstens! I hear we are about to check up on your labs for the surgery.”

*Turns to the intern:* “And you must be our new intern! We haven’t met yet, my name is Dr. Müller. I don’t know Mr. Karstens very well, can you please give me a detailed handover about his current situation?”

**Scenario 2**

*Nurse approaches the patient to check his vital signs.*

*Nurse:* “You are the intern, who was supposed to draw blood right? I just came in and Mr. Karstens told me, that he is not feeling well today.”

*Checkbox: What would you like to do first?*

1. Ask the nurse to tell you the vital signs she just took
2. Take a detailed patient history
3. Draw the blood sample

- *All answers are correct, the scenario continues accordingly until all buttons have been clicked.*

*Nurse:* “The vitals are normal, except for mild tachycardia.”

*Vital signs are visible above the patient: P 100/min, sO2 96%, RR 110/60mmHg, T 36.9°C*

*Patient:* “I have already told the nurse, I am not feeling great today. I am so tired and I get winded after a couple of steps. It’s not usually like that. I am not in pain, but this morning I noticed my stools to be black, I have never seen anything like this before.”

*Checkbox: The lab called hemoglobin levels of Mr. Karstens have decreased from 14.1mg/dl to 12.3mg/dl within the last three days. What are the next steps you would like to take?*

1. Watchful waiting, ask the patient to let you know if anything changes.
2. Check on clotting factors in the lab, consider to draw blood for blood typing, consider endoscopy
3. Call the emergency team, emergency CT-scan, transfer the patient to ICU

- *b) is the correct answer*

*Checkbox: Are there any other consequences you would draw from these findings?*

1. No other consequences, surgery can take place, since hemoglobin of 12.3mg/dl is sufficient.
2. Prioritize surgery endoscopy can take place simultaneously.
3. Hernia repair surgery should be postponed since there is a suspicion of gastrointestinal bleeding, which is a more pressing issue.

- *c) is the correct answer*

*Nurse:* “The attending will be here any minute, maybe you can talk to him about the situation.”

*Attending enters and greets everybody.*

*Attending:* “Good Morning Mr. Karstens! I hear we have already taken a lab sample to make sure everything is in order for your surgery?”

*Turns to the intern:* ““And you must be our new intern! We haven’t met yet, my name is Dr. Müller. I don’t know Mr. Karstens very well, can you please give me a detailed handover about his current situation?”

**Scenario 3**

*The patient looks unwell and has seemingly passed out. The nurse approaches and talks loudly to the patient while palpating his pulse.*

*Nurse:* “Mr. Karstens, open your eyes please, can you hear me?”

*Turns to the intern:* “I just came in and found him like this. Earlier he said, he was dizzy, now he is barely reacting. His pulse feels high and he feels clammy.”

*Checkbox: In which order would you like to proceed?*

1. Check vital signs
2. Take a patient history
3. Draw a blood sample
4. Check on vigilance, breathing and pulse

- All answers are correct, the scenario continues accordingly until all buttons have been clicked.

*Vital signs are visible above the patient: P 120/min, sO2 95%, RR 70/40mmHg, T 36.9°C*

*Nurse:* “I will check breathing and pulse. Pulse is palpable over 100 for sure, airway is not obstructed, but his breathing is shallow and accelerated.”

*Patient (mumbling):* “I don’t feel good, I am so tired… also I think I wet the bed, everything is moist…”

*Nurse:* “I will draw a blood sample and bring it to the lab.”

*The nurse removes the covers, now it becomes apparent, that the bed is soaked in bloody stool.*

*Turns to the intern:* “I will call help now, while you can take first measures on the patient. Who should I call first?”

*Checkbox: Who should the nurse call?*

1. Attending
2. Emergency team
3. Both first attending then emergency team
4. Both first emergency team then attending

- d) is the correct answer

*Nurse:* “They will be here any minute!”

*Emergency team arrives, shortly after the attending arrives and ask the intern for a handover.*
