# Supplementary material for: Use of an Immersive Virtual Reality Application to Educate Medical Students in Patient Handover: Pilot Study
Source: JMIR Serious Games. 2025 Aug 27;13:e73907. doi: 10.2196/73907 (PMC12384679; doi:10.2196/73907)
Supplement: Multimedia Appendix 2 [file games-v13-e73907-s002.docx]

Questionnaires for the manuscript with the title “Use of an immersive virtual reality application to educate medical students in patient handover”

**Questionnaire 1: Participants after VR training**

1. How old are you?
2. Gender: How do you identify?
   1. Male
   2. Female
   3. Diverse
3. Do you wear glasses/contact lenses?
   1. Yes
   2. No
4. If you answered “yes” in 3., did you wear your glasses/contact lenses?
   1. Yes
   2. No
5. Did you complete any professional training before studying medicine?
   1. Yes
   2. No
6. If you answered “yes” in 5., please specify!
7. Have you worked in the medical field before?
   1. Yes
   2. No
8. If you answered “yes” in 7., please specify!
9. Have you ever taken part in a curriculum or course in patient handover before the surgical practical course?
   1. Yes
   2. No
10. Did you feel secure and confident in the topic of handover **before** training in VR?

1 2 3 4 5 6 7

Very confident **O O O O O** **O O** very inconfident

1. Did you feel secure and confident in the topic of handover **after** training in VR?

1 2 3 4 5 6 7

Very confident **O O O O O** **O O** very inconfident

1. Have you used VR equipment or head mounted displays in the past?
   1. Never
   2. Less than 5 times
   3. More than 5 times
   4. More than 10 times
2. Did you feel unwell, dizzy or motion sick during the training in VR?

Not at all very much

**0**---1---2---3---4---5---6---7---8---9---**10**---11---12---13---14---15---16---17---18---19---**20**

1. Did you have discomfort or pain in your eyes, did your eyes water a lot or did they feel strained?

Not at all very much

**0**---1---2---3---4---5---6---7---8---9---**10**---11---12---13---14---15---16---17---18---19---**20**

Questions 15 – 23: Question of the immersion scale after Nichols in German [1]

1. Do you think virtual reality equipment can be a useful addition to medical school curricula or for other medical professions?

1 2 3 4 5 6 7

Very useful **O O O O O** **O O** not useful at all

1. Did you have fun during the handover training in virtual reality?

1 2 3 4 5 6 7

More fun **O O O O O** **O O** less fun than in other courses

than in other

courses

1. Nichols, S., C. Haldane, and J.R. Wilson, *Measurement of presence and its consequences in virtual environments.* International Journal of Human-Computer Studies, 2000. **52**(3): p. 471-491.

**Questionnaire 2: Participants after OSCE**

1. How confident did you feel regarding the learning objectives of the station “handover”?

1 2 3 4 5 6 7

Very confident **O O O O O** **O O** very inconfident

1. How confident did you feel during the OSCE?

1 2 3 4 5 6 7

Very confident **O O O O O** **O O** very inconfident

1. Looking back, did you find the training in virtual reality helpful for exam preparation?

1 2 3 4 5 6 7

Very helpful **O O O O O** **O O** not helpful at all

1. Do you think virtual reality equipment can be a useful addition to medical school curricula or for other medical professions?

1 2 3 4 5 6 7

Very useful **O O O O O** **O O** not useful at all

1. Would you like to use virtual reality equipment regularly to learn new skills?

1 2 3 4 5 6 7

Strongly disagree **O O O O O** **O O** strongly agree

1. Do you think virtual reality equipment can be used to replace contact to patients i.e. during social distancing measures like during the COVID-19 pandemic.

1 2 3 4 5 6 7

Strongly disagree **O O O O O** **O O** strongly agree

1. Do you think virtual reality equipment can be used as a meaningful addition to patient contact?

1 2 3 4 5 6 7

Strongly disagree **O O O O O** **O O** strongly agree

1. Were you able to remember well what you learned during the surgical practical course and were you able to use this knowledge during the OSCE?

1 2 3 4 5 6 7

Very good **O O O O O** **O O** very bad

1. Did you have fun studying for the station handover?

1 2 3 4 5 6 7

Yes a lot **O O O O O** **O O** none at all

1. How much time did you spend studying for the handover station?

1 2 3 4 5 6 7

A lot **O O O O O** **O O** almost none

1. How high was your motivation to study for the handover station?

1 2 3 4 5 6 7

Very high **O O O O O** **O O** very low

1. Do you have any additions?

Free text.

**Questionnaire 3: Whole semester after OSCE**

1. How old are you?
2. Gender: How do you identify?
   1. Male
   2. Female
   3. Diverse
3. Do you wear glasses/contact lenses?
   1. Yes
   2. No
4. If you answered “yes” in 3., did you wear your glasses/contact lenses?
   1. Yes
   2. No
5. Did you complete any professional training before studying medicine?
   1. Yes
   2. No
6. If you answered “yes” in 5., please specify!
7. Have you worked in the medical field before?
   1. Yes
   2. No
8. If you answered “yes” in 7., please specify!
9. Have you ever taken part in a curriculum or course in patient handover before the surgical practical course?
   1. Yes
   2. No
10. How much time did you spend studying for the handover station?

1 2 3 4 5 6 7

A lot **O O O O O** **O O** almost none

1. How confident did you feel during the handover OSCE?

1 2 3 4 5 6 7

Very confident **O O O O O** **O O** very inconfident

1. Were you able to remember well what you learned during the surgical practical course and were you able to use this knowledge during the OSCE?

1 2 3 4 5 6 7

Very good **O O O O O** **O O** very bad

1. Did you have fun studying for the station handover?

1 2 3 4 5 6 7

Yes a lot **O O O O O** **O O** none at all

1. How high was your motivation to study for the handover station?

1 2 3 4 5 6 7

Very high **O O O O O** **O O** very low
